# A spot laser modulated resistance switching effect observed on n-type Mn-doped ZnO/SiO_2_/Si structure

**DOI:** 10.1038/s41598-017-15556-6

**Published:** 2017-11-09

**Authors:** Jing Lu, Xinglong Tu, Guilin Yin, Hui Wang, Dannong He

**Affiliations:** 1 National Engineering Research Center for Nanotechnology, No. 28 East Jiangchuan Road, Shanghai, 200241 P.R. China; 2 School of Materials Science and Engineering, Shanghai Jiao Tong University, No. 800 Dongchuan Road, Shanghai, 200240 P.R. China; 3 School of Physics and Astronomy, Shanghai Jiao Tong University, No. 800 Dongchuan Road, Shanghai, 200240 P. R. China

## Abstract

In this work, a spot laser modulated resistance switching (RS) effect is firstly observed on n-type Mn-doped ZnO/SiO_2_/Si structure by growing n-type Mn-doped ZnO film on Si wafer covered with a 1.2 nm native SiO_2_, which has a resistivity in the range of 50–80 Ω∙cm. The I–V curve obtained in dark condition evidences the structure a rectifying junction, which is further confirmed by placing external bias. Compared to the resistance state modulated by electric field only in dark (without illumination), the switching voltage driving the resistance state of the structure from one state to the other, shows clear shift under a spot laser illumination. Remarkably, the switching voltage shift shows a dual dependence on the illumination position and power of the spot laser. We ascribe this dual dependence to the electric filed produced by the redistribution of photo-generated carriers, which enhance the internal barrier of the hetero-junction. A complete theoretical analysis based on junction current and diffusion equation is presented. The dependence of the switching voltage on spot laser illumination makes the n-type Mn-doped ZnO/SiO_2_/Si structure sensitive to light, which thus allows for the integration of an extra functionality in the ZnO-based photoelectric device.

## Introduction

Resistance switching (RS) effect refers to reversible changes between two or more metastable states induced by applying an external voltage. As one of the most significant paradigm among existing resistance manipulation methods, it has attracted much attention due to the application potentials in next generation of non-volatile memory devices^1–4^. Therefore, intense effort has been made to explore excellent RS materials. Various kinds of materials including ferromagnetic materials^5^, organic materials^6,7^, perovskites^8,9^ and binary metal oxides^10–14^ were extensively studied. Among these candidates, binary metal oxides have great advantages owning to their simple structure, easy fabrication process and compatibility with metal-oxide semiconductor (MOS) devices^10,15–18^. As an important binary transition metal oxide, ZnO has been widely applied in electronics, optics, optoelectronics, spintronics, thin film transistors and so on because of its adjustable electrical, optical, even magnetic properties and facile preparations^19–24^. According to previous reports, ZnO host material has exhibited high performance on RS effect based on applying traditional electrical field as single stimulation^14,15,25^. However, with the quick development physical limits are gradually approached under single stimulation. As a result, compound stimulations are used to manipulate RS effect in ZnO based materials^26–30^. However, using spot laser as co-existing stimulation has been scarcely reported.

In this paper, we give a systematic study on spot laser modulated resistance switching effect, which is firstly observed in a rectifying junction of n-type Mn-doped ZnO/SiO_2_/Si structure. By introducing a laser beam as an extra stimulation, the switching voltage, which drives the resistance state of the structure switch from one state to the other, shows clear shift with the change of spot laser illumination position on either the *n*-type Mn-doped ZnO film or Si substrate surface. Moreover, the switching voltage shift also shows dependence on the spot laser power. We ascribe the dual dependence to the electrical field produced by the redistribution of photo-generated carriers under spot laser illumination. The produced field enhances the internal barrier of the hetero-junction and thus induces the switching voltage shift. This light sensitive character in the proposed n-type Mn-doped ZnO/SiO_2_/Si structure adds a new parameter in RS controlling methods, which makes the structure an outstanding candidate for integrated multi-functional photoelectric device.

## Methods

The structure is vertical stacks with the n-type Mn-doped ZnO film grown on Si wafer. The (111) Si wafer was of 0.3 mm thick, which had a resistivity in the range of 50–80 Ω∙cm, covered with thin native SiO_2_ of 1.2 nm. The n-type Mn-doped ZnO film was deposited by co-coping specially designed Al-doped ZnO ceramic (composed of 2% Al_2_O_3_ and 98% ZnO) and Mn metallic (99.99%) targets at room temperature. The base vacuum of the chamber was better than 4.5 × 10^−5^ Pa prior to deposition and the working argon pressure of 0.85 Pa was maintained during deposition. The dopant concentration of Mn was controlled by the DC magnetron sputtering power of Mn metallic target when the radio frequency power of the ceramic target was maintained at 50 W. The alloying indium electrode for electrical measurement, which showed no measurable rectifying behaviour, had a diameter less than 1 mm. All current-voltage (I–V) characteristics were measured by Keithley-4200 semiconductor characterization system. During the sweeping process, the sweeping rage is 6 s/curve with 0.1 V step, which would be obtained in all measurements. The optical transmittance spectra of the deposited films were determined by UV-Vis-NIR spectrophotometer in range of 200–1000 nm on glass substrate. The spot laser used in the study is from a 532 nm laser focused into a roughly 50 µm-diameter, whose output optical power ranges between 0 and 10 mW through an optical attenuation. A light emitting diode (LED) is used as light source to avoid any influence of heat transfer into the sample.

## Results and Discussion

**Figure 1:**
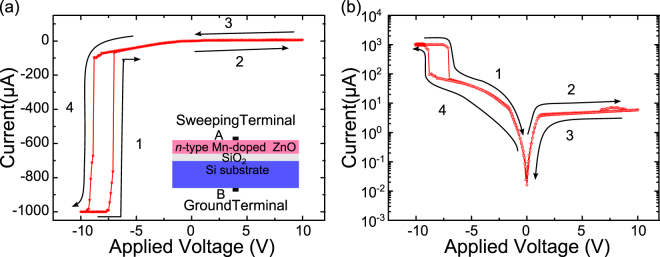
I–V characteristics of the n-type Mn-doped ZnO/SiO_2_/Si structure in linear (**a**) and semi-log (**b**) patterns. The arrows indicate the sweeping operation sequence. The inset displays the schematic illustration of the measurement mode.

In Fig. 1 we present the typical I–V characteristic of the proposed structure measured in dark condition (without illumination) at room temperature in linear (a) and semi-log (b) patterns, separately. The voltage is swept in four parts following a sequence marked in Fig. 1 adopting a current compliance of 1 mA herein to avoid permanent dielectric breakdown of the device. The structure is initially in a low resistive state (LRS) under −10V negative bias, while a voltage above a critical value (about −7V) switches it to a high resistive state (HRS). There exists a significant difference of the output current in two states, corresponding to ON/OFF state, which is a capacitive induced phenomenon and working principle of thin film transistors^18,22–24^. For forward bias, which corresponds to a positive sweeping voltage applied to the electrode A, only a small current is observed independent of the voltage value. This I–V curve evidences the prepared structure a rectifying junction. With respect to the inverse sweeping process (part 3 and 4), the LRS is read at a voltage of about −9V. According to polarities of applied voltages, this RS behaviour was categorized into unipolar type, which was a novel phenomenon for memory material grown on Si substrate^4^.

**Figure 2:**
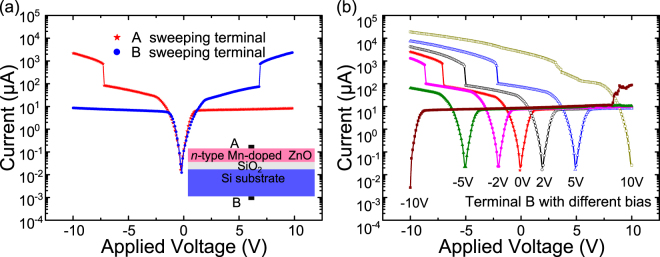
I–V characteristics of the n-type Mn-doped ZnO/SiO_2_/Si structure measured with Electrodes connected in inverse modes (**a**) and Electrode B placed at different bias voltages. Current values are represented absolute ones.

To exhibit the polarity of RS effect obtained in the prepared structure, we further exploit the I–V characteristic curves of the junction adopting a compliance of 0.1 A to record actual current value. As is shown in Fig. 2(a), the I–V curves measured in inverse mode show axial symmetry property. When the sweeping electrode A (demonstrated as inset) connected as ground terminal, the switching voltage is reversed to 7V from −7V correspondingly, implying the electric field driven origin. For further verification, we also re-measured I–V curves with placing electrode B at different bias, displayed as Fig. 2(b). For simplicity, all curves presented in following were measured with electrode A connecting up sweeping voltage without specific caption. As is shown in Fig. 2(b), the switching voltage presents linear shift with the bias within limited range owing to the linear offset of the bias and sweeping voltage. Meanwhile, the offset linearity deteriorated significantly with a high bias voltage (either positive or negative), which also suggested the junction current characteristic of the structure.

When the spot laser was introduced as co-stimulation to modulate the RS effect, the switching voltage shows a dual dependence on both the illumination position and the laser power. To investigate this dual influence, we re-measured the I–V characteristic curves of the prepared structure in two different designed modes, defined as CP (constant power) mode and CD (constant distance between the illuminating position and the electrode) mode, respectively.

**Figure 3:**
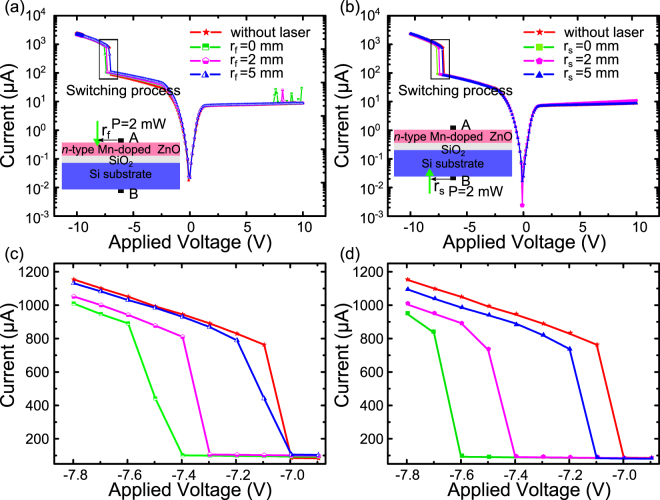
I–V characteristics of the structure with a spot laser (P = 2 mW) illuminating on different surface positions of (**a**) n-type Mn-doped ZnO film (**b**) Si Substrate surface. Enlarged view of switching process with laser illuminating on film surface (**c**) and (**d**) substrate.

Figure 3 presents the I–V curves response to spot laser illuminating different positions on both Si substrate and film surface in CP mode (with a 2 mW output laser power). As is shown in Fig. 3(a,b), the switching voltage varies obviously as the spot laser illumination moves away from the electrode position. To display the influence of illumination position on the switching voltage clearly, the enlarged views of the current response to the voltage during the switching process are exhibited in Fig. 3(c,d), respectively. Compared to the switching voltage need without illumination, the switching voltage shift reaches highest as the spot laser illuminates nearby the electrode. On film side, the switching voltage shifts to −7.4V from −7V. With the increase of the distance between the illumination position and electrode, the switching voltage shift becomes less and less and finally is negligible once the distance reaches a threshold value. On film side, the switching voltage returns back to −7V with a 5 mm distance, indicating a threshold value of 5mm. By contrast, there is a little difference on the substrate side. When the spot laser illuminated at the position of 5mm away from the electrode, the switching voltage is −7.1V, which remains away from the initial value. We ascribe this to the different influence of the photo-generated carrier in two sides, which would be explicated in detail later.

**Figure 4:**
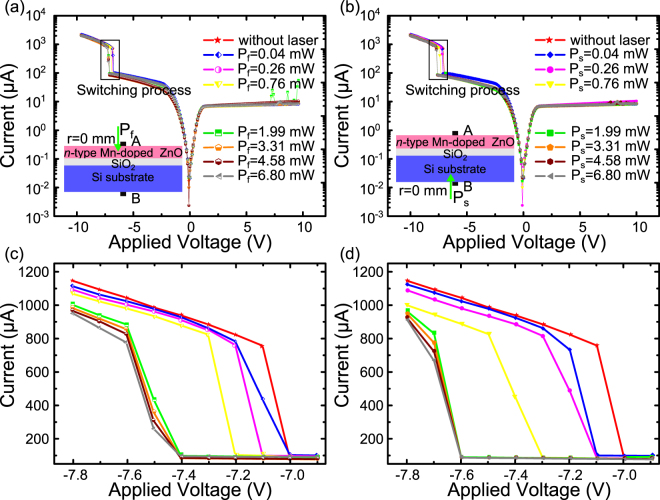
I–V characteristics of the structure with a spot laser of different powers illuminating on same positions (r = 0 mm) of (**a**) n-type Mn-doped ZnO film (**b**) Si Substrate. Enlarged view of switching process with laser illuminating on film surface (**c**) and (**d**) substrate.

In Fig. 4, we give the I–V curves response to different power laser illumination in CD mode, in which the laser illuminating position was fixed nearby the electrodes on both film and Si substrate side. The output optical power ranges from 0 to 6.8 mW, which is modulated through an optical attenuation. As is shown in Fig. 4(a,b), the switching voltage shift becomes more and more obvious on both film and Si substrate with the increasing laser power and reaches highest under a 2 mW laser illumination. On film side the highest switching voltages is −7.4V, and on Si substrate it reaches −7.6V. As the illumination laser power exceeds 2 mW, the switching voltage stops shifting and becomes statured on both film side and Si substrate, as is shown in Fig. 4(c,d). On the film side the switching voltage keeps at −7.4V constantly. In the same condition, the constant switching voltage is kept at −7.6V on Si substrate side. Noticeably, there exists a slight degree of difference during the switching process as the laser power exceeds the saturated value. The proposed structure has switched to high resistance state with laser illuminating on film side. By contrast it tends to remain in low resistance state on Si substrate.

The mechanism behind RS has been controversial for a long time. Now there is consensus on conducting filament model due to the direct evidence given by the high-resolution conducting atomic force microscope (CAFM)^7,11,31^. Here we just focus on the dual dependence of the switching voltage on the position and power of the illuminating laser, which is ascribed the offset effect of enhanced internal barrier of the hetero-junction induced by the redistribution of photo-generated electron-hole pairs overlapped on the switching voltage.

**Figure 5:**
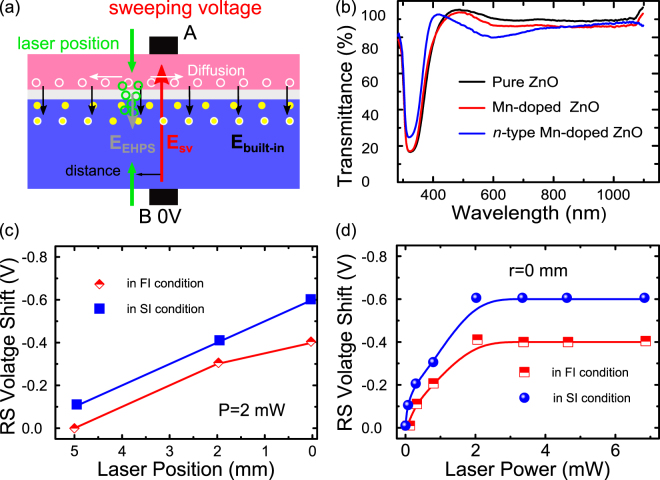
(**a**) The general schematic mechanism of laser illumination influence on the switching voltage in n-type Mn-doped ZnO/SiO_2_/Si structure. (**b**) Optical transmission spectra of n-type Mn-doped ZnO film, Mn-doped film and ZnO film. (**c**,**d**) The Switching Voltage shift dependence on laser position and power in FI and SI condition.

As is shown in Fig. 5(a), a local electric filed opposite to the built-in electric field is formed during the switching process (marked as E_SV_) between the electrodes. Without laser illumination, the switching voltage overcomes the built-in field (marked as E_built-in_) and drives the structure to a different resistance state by forming localized conducting filaments^32^. When the prepared structure is illuminated by the spot laser either on the film or on the Si substrate nearby the electrode, most of photos would be absorbed by the Si substrate due to the high transmittance of the film, which is well confirmed by the transmittance spectra of the *n*-type Mn-doped ZnO film displayed in Fig. 5(b), as well as the Mn-doped and pure ZnO films. The absorption produces electron-hole pairs in a restricted region in Si substrate equal to the irradiated area, which diffuse to junction where they are separated by the electric force. The holes are swept into the films and the electrons remain in substrate side. This process produces a photo voltage at the illumination position, exhibiting as an extra electric field (marker as E_EHPS_) in the same direction with built-in field, which therefore enhances the internal barrier. This photo voltage V_EHPS_, representing the added internal barrier generated by the electron-hole pairs, is related to the number of electron-hole pairs separated by the junction by the following relationship:

1 $${V}_{EHPS}=(\frac{kT}{q})\mathrm{ln}(\frac{qf}{{J}_{S}}+1)$$

where q is the magnitude of the electronic charge, k is Boltzmann’s constant and T is the absolute temperature. And J_S_ is the saturation current. Noticeable here the spot laser is monochromatic, so the number of photo generated holes (electrons) *f* in the illumination area can be written as:

2 $$f(0)=\frac{P\xi T}{hv}$$

here P is the incident laser power, ξ is the quantum efficiency and T represents the transmittance of laser to the film. Quantized photon energy is described as hν according to quantum theory (h is the Planck’s constant and ν is the laser frequency).

As the laser illuminates away from the electrode, the photo generated holes would diffuse along the interface from the illumination position, shown as Fig. 5(a). Thus the photo voltage generated by the excess holes approaching the area of E_SV_ would become as follow:

3 $${V}_{EHPS}(r)=(\frac{kT}{q{J}_{S}}){f}_{{\rm{0}}}\exp (-\frac{r}{{r}_{0}})=\frac{P\xi T}{hv}(\frac{kT}{q{J}_{S}})\exp (-\frac{r}{{r}_{0}})$$

where r represents the distance between the illumination position and electrode, r_0_ is the diffusion length of the photo generated carriers along the interface. The process is similar to the case calculated in ref.^33^.

To verify the relation between the switching voltage shift and laser position and power, the dependence of switching voltage shift on the laser position and power are shown as Fig. 5(c,d). As is shown, the switching voltage shift presents a quasi-linear dependence on the distance between the electrode and the laser position within 5 mm in both FI (film illuminated) and SI (substrate illuminated) condition, displayed in Fig. 5(c). The better linearity and remaining effect in SI condition with r = 5 mm is ascribed to fewer defects existing in Si substrate compared to the *n*-type Mn-doped film. Besides, the dependence of switching voltage on laser power in FI and Si condition displayed in Fig. 5(d), clearly exhibits the raise of switching voltage shift with the increasing laser power at first, and then gets saturated as the laser power exceeds the threshold value. However, the switching shift observed in SI condition is much larger in CP mode than in FI condition. This is a result of the total absorption of photons in Si substrate in SI condition. The total absorption generates more electron-hole pairs, having a larger influence on the switching voltage shift according to eq. (3).

In summary, a spot laser modulated RS effect is firstly observed on n-type Mn-doped ZnO film/SiO_2_/Si structure, in which the Si substrate has a resistivity in the range of 50–80 Ω∙cm with a native SiO_2_ layer. The prepared structure works as a rectifying junction, in which the change of resistance is a capacitive induced phenomenon. By combining spot laser illumination as co-existing stimulation, the switching voltage varies with both laser illumination position and power, which adds a completely new degree of freedom of RS modulation. Based on the redistribution of photo-generated electron-hole pair’s mode, the added internal barrier induced by these carriers is proposed to account for the dual dependence of switching voltage on laser position and power. These achievements suggest a novel approach to improve the RS effect performance. The light sensitive character also makes n-type Mn-doped ZnO/SiO_2_/Si structure an excellent candidate for multi-functional photoelectric device in RS based storage technology.

## Electronic supplementary material

Supplementary information
