# Supplementary material for: A spot laser modulated resistance switching effect observed on n-type Mn-doped ZnO/SiO2/Si structure
Source: Sci Rep. 2017 Nov 9;7:15221. doi: 10.1038/s41598-017-15556-6 (PMC5680311; doi:10.1038/s41598-017-15556-6)

A spot laser modulated resistance switching effect observed on *n*-type Mn-doped $ZnO/SiO_2/Si$ structure

Supplementary information

Jing Lu, [1] Xinglong Tu, [1,2] Guilin Yin, [1,2] Hui Wang [3,*)] and Dannong He[1,2,*)]

[1] National Engineering Research Center for Nanotechnology, No.28 East Jiangchuan Road, Shanghai, 200241, P.R. China

[2] School of Material Science and Engineering, Shanghai Jiao Tong University, No.800 Dongchuan Road, Shanghai 200240, PR China

[3] School of Physics and Astronomy, Shanghai Jiao Tong University, No.800 Dongchuan Road, Shanghai, 200240, P. R. China

*Corresponding author:

huiwang@sjtu.edu.cn and hdn_nercn@163.com

The evidence that the pre-prepared ZnO based film is *n*-type Mn-doped ZnO film.

Figure S1 SEM images for n-type Mn-doped ZnO film, the Mn target maintained at 5W during deposition process

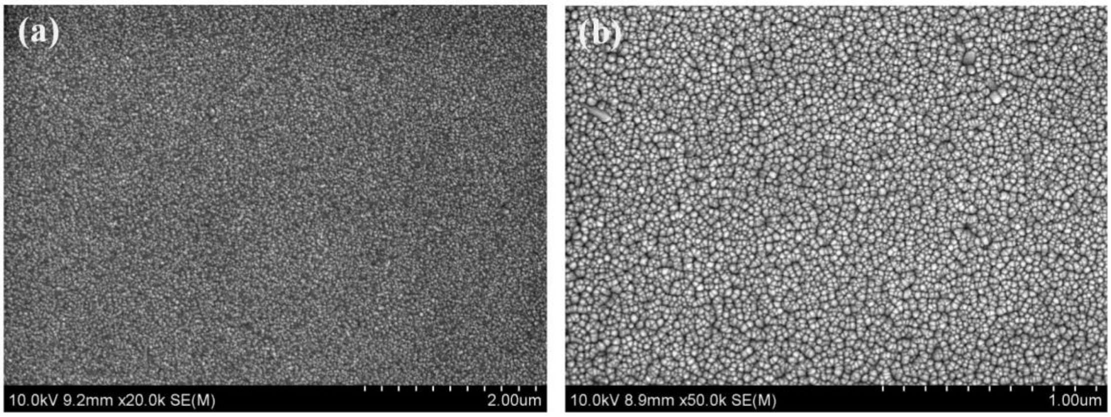

Figure S2 EDS elemental mapping results for area shown in Figure S1(a), showing that Al and Mn are well co-doped in ZnO in n-type Mn-doped ZnO film preparation.

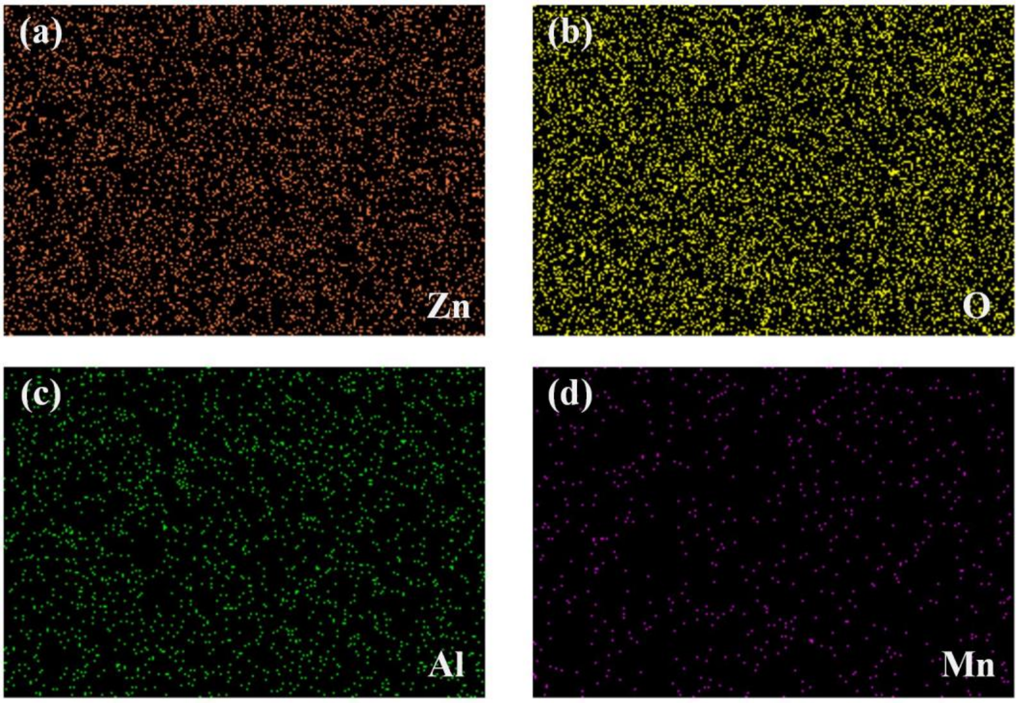

Supplement: Supplementary file 1 — Supplementary information [file 41598_2017_15556_MOESM1_ESM.pdf]
